# Contribution of transformation products towards the total herbicide toxicity to tropical marine organisms

**DOI:** 10.1038/s41598-018-23153-4

**Published:** 2018-03-19

**Authors:** Philip Mercurio, Geoff Eaglesham, Stephen Parks, Matt Kenway, Victor Beltran, Florita Flores, Jochen F. Mueller, Andrew P. Negri

**Affiliations:** 1 Australian Institute of Marine Science, Townsville, Queensland Australia; 2 The University of Queensland, Queensland Alliance for Environmental Health Sciences, Coopers Plains, Queensland Australia; 3 James Cook University, Townsville, Queensland Australia

## Abstract

The toxicity of herbicide degradation (transformation) products is rarely taken into account, even though these are commonly detected in the marine environment, sometimes at concentrations higher than the parent compounds. Here we assessed the potential contribution of toxicity by transformation products of five photosystem II herbicides to coral symbionts (*Symbiodinium* sp.), the green algae *Dunaliella* sp., and prawn (*Penaeus monodon*) larvae. Concentration-dependent inhibition of photosynthetic efficiency (*∆F/F*_*m*_′) was observed for all herbicides in both microalgal species. The toxicity of solutions of aged diuron solutions containing transformation products to *Symbiodinium* sp. and *Dunaliella* sp. was greater than could be explained by the concentrations of diuron measured, indicating transformation products contributed to the inhibition of *∆F/F*_*m*_′. However, the toxicity of aged atrazine, simazine, hexazinone, and ametryn solutions could be explained by the concentration of parent herbicide, indicating no contribution by transformation products. Prawn larval metamorphosis was not sensitive to the herbicides, but preliminary results indicated some toxicity of the transformation products of atrazine and diuron. Risk assessments should take into account the contribution of herbicide transformation products; however, further studies are clearly needed to test the toxicity of a far wider range of transformation products to a representative diversity of relevant taxa.

## Introduction

Tropical marine ecosystems are highly diverse and support many vulnerable and protected species including corals, seagrass, dugong, and sea turtles. In tropical Queensland Australia, these sensitive marine environments adjoin considerable areas of agriculture, which represents a source of pollutants that contribute to the decline of water quality and reef health of the World Heritage-listed Great Barrier Reef (GBR)^1^. Agricultural herbicides are desinged to kill weeds, and their high water solubility and mobility has led to contamination of nearshore marine environments, including the GBR^2,3^. Photosystem II (PSII) herbicides are the most widely detected group of herbicides in marine ecosystems and these act to block photosynthetic electron transport in weeds, but also inhibit photosynthesis in native marine plants and algae. The chronic exposure of sensitive environments including wetlands, estuaries, seagrass beds, and coral reefs to PSII herbicides following monsoonal flood events is of particular concern^4^. The risks of long-term exposures are likely as PSII herbicides have been detected in marine and estuarine systems year-round^5,6^, and this is at least partly due to their long persistence in seawater of >100 d^7,8^.

Herbicides can harm tropical marine organisms including coral^9–12^, isolated coral symbionts (*Symbiodinium* sp.)^13,14^, microalgae^15,16^, and seagrass^17–21^. However, few herbicide toxicity studies have considered their transformation (degradation) products, even though these are regularly detected in the environment, and sometimes at concentrations higher than the parent herbicide^22–25^. The transformation products of the PSII herbicides diuron and atrazine have been frequently reported in the GBR region^26,27^, sometimes reaching concentrations over 2 μg l^−1^ ^28^. The transformation products can exert similar acute and chronic toxicities as the parent compound^29^, and a review by Sinclair and Boxall (2003)^24^ revealed that 30% of herbicide transformation products are more potent than the parent compound. When the toxicities of herbicides and their transformation products are combined, the total toxicity can increase by up to an order of magnitude^22^. One of diuron’s main transformation product 3,4-dichloroaniline (3,4-DCA) has been reported to be more toxic to some species than its parent compound^30–33^. Atrazine’s primary environmental transformation products, desethylatrazine (DEA) and desisopropylatrazine (DIA), are reported to have similar toxicities to atrazine^34,35^. However, microalga common in tropical estuaries were less sensitive to 3,4-DCA and DEA than their parent herbicides^16^, adding to the ambiguity regarding contributions of herbicide transformation products to total toxicity^36^.

The combined contributions of parent compounds and transformation products have been included in some overall risk assessments^37–39^, but this is not a commonly applied approach. When the toxic mechanism of herbicide and transformation products are the same, chemical addition (total toxicity mixture = ∑C_*i*_ × P_*i*_, where C_*i*_ is the concentration of herbicide *i* and P is the potency of herbicide *i* relative to the reference herbicide) can be used to derive total mixture toxicities^16,40,41^. However, this approach cannot account for (i) transformation products of unknown herbicidal toxicity, (ii) transformation products that have not been identified, and (iii) transformation products with different toxic mechanisms that may affect other non-target species such as animals. In addition, the persistence of toxic transformation products may be long and contribute to chronic ecological risk^36,42^.

Transformation products should be incorporated into water quality guidelines and chemical risk assessments^43^ but specific data on toxicities of the myriad of potential transformation products is not available^36^. In order to evaluate the potential contribution of toxicity by known and unknown transformation products of five PSII herbicides we compared the acute toxicity of partially aged PSII herbicides (including transformation products) with their parent compounds^36^. The aged herbicides were generated over 330 days in 120 l outdoor tanks containing natural coastal seawater and sediments (see Methods). The toxicities of parent and aged pesticides were compared using relevant tropical marine photosynthetic organisms (i) the coral symbiont (*Symbiodinium* sp.) and (ii) the green algae *Dunaliella* sp. and a non-photosynthetic organism (iii) prawn (*Penaeus monodon*) larvae.

## Results

### Toxicity of aged herbicide solutions to microalgae

**Figure 1:**
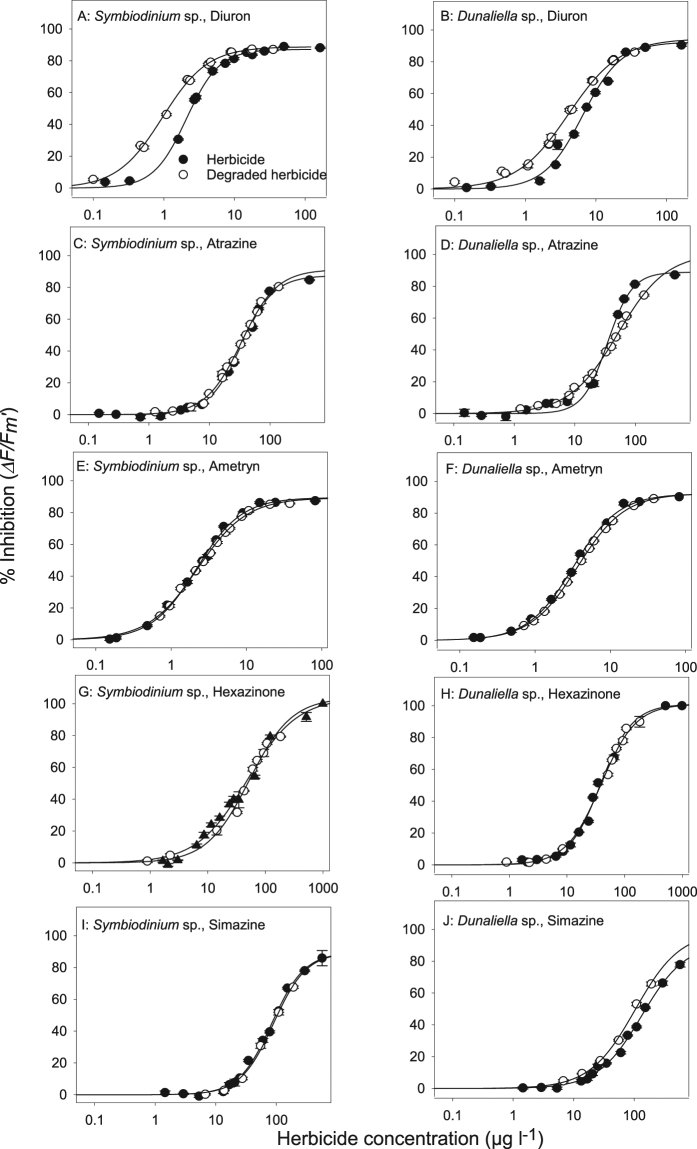
Concentration-response curves for herbicides and their transformation products to microalgae. Inhibition of *∆F/F*_*m*_′ (% relative to control) for *Symbiodinium* sp. and *Dunaliella* sp. for both herbicide and aged herbicides (the concentrations of transformation products are not considered here). Overlapping concentration-response curves indicate similar toxicities (Table 1).

Concentration-dependent inhibition of *∆F/F*_*m*_*′* by all herbicide solutions were observed for both microalgal species (Fig. 1). The concentrations of herbicide inhibiting *∆F/F*_*m*_*′* by 10%, 20% and 50% (IC_10_ IC_20_ and IC_50_ respectively) were calculated for the parent herbicides and aged herbicide solutions containing transformation products (based on the measured concentrations of parent herbicides) (Tables 1 and 2). The composition of aged solutions for all concentrations used in the microalgae toxicity tests are outlined in SOM Table 5. Apart from diuron, the toxicity in all aged herbicide mixtures could be explained by the concentrations of the parent herbicide (e.g. the IC_10_s, IC_20_s, and IC_50_s between parent and aged herbicides were not significantly different) (Table 1). In contrast, all IC_X_s of the aged diuron solutions were lower for *Symbiodinium* sp., as were the IC_10_s and IC_20_s for *Dunaliella* sp., indicating additional contribution to toxicity by transformation products.

**Table 1:** Comparison of IC_X_ (µg l^−1^) values of standard parent herbicides and aged mixtures (after 330 d) from each herbicide. These IC_X_ values represent the concentration of parent herbicide in the toxicity assays that inhibit *∆F/F*_*m*_′ by X = 10, 20 or 50% and were derived from the concentration-response curves in Fig. 1 (the concentrations of transformation products are not considered here). Differences in IC_X_s between pure and aged herbicide solutions were assessed using the F-test in GraphPad and were considered significant when < 0.05. The diuron vs aged diuron solutions had significantly different IC_10_ and IC_20_ values for both algal species and the IC_50_ values for *Symbiodinium sp*. were different. IC_10_ and IC_20_ values for all herbicide and algal species can be found in Table 2. All r^2^ > 0.97.

	IC_X_	IC_X_ parent	IC_X_ aged	F(df)	P value
*Symbiodinium sp*.					
Diuron	IC_50_	1.4 (1.3–1.6)	0.95 (0.92–0.99)	F (1,84) = 34.1	<0.0001
	IC_20_	0.68 (0.58–0.79)	0.34 (0.32–0.36)	F (1,84) = 74.3	<0.0001
	IC_10_	0.64 (0.57–0.71)	0.17 (0.12–0.24)	F (1,84) = 136	<0.0001
Atrazine	IC_50_	34.5 (32–35)	32 (27–38)	F (1,110) = 0.352	0.5541
Ametryn	IC_50_	2.2 (2.1–2.3)	2.3 (2.2–2.4)	F (1,93) = 2.13	0.1475
Hexazinone	IC_50_	45.7 (40–53)	51 (46–56)	F (1,89) = 3.41	0.068
Simazine	IC_50_	84 (77–92)	72.5 (63–83)	F (1,74) = 1.59	0.2111
*Dunaliella sp*.					
Diuron	IC_50_	4.4 (4.2–4.6)	4.1 (3.8–4.5)	F (1,103) = 0.980	0.3245
	IC_20_	1.74 (1.65–1.84)	1.27 (1.19–1.34)	F (1,103) = 62.1	<0.0001
	IC_10_	1.02 (0.94–1.10)	0.63 (0.58–0.69)	F (1,103) = 63.4	<0.0001
Atrazine	IC_50_	35 (32–38)	40 (35–47)	F (1,95) = 1.96	0.1652
Ametryn	IC_50_	3.4 (3.2–3.6)	3.7 (3.5–3.8)	F (1,81) = 17.3	0.0789
Hexazinone	IC_50_	38 (36–40)	40 (38–43)	F (1,87) = 2.24	0.1384
Simazine	IC_50_	87 (79–96)	103 (73–146)	F (1,78) = 0.841	0.3618

**Table 2:** Comparison of IC_10_ and IC_20_ (µg l^−1^) values of herbicides, aged herbicides and DEA, the transformation product of atrazine for *Symbiodinium* sp. and *Dunaliella* sp. These IC_X_ values represent the concentration of parent herbicide in the toxicity assays that inhibit *∆F/F*_*m*_′ by X = 10 and 20% and were derived from the concentration-response curves in Fig. 1. Of the three transformation products tested, only DEA inhibited *∆F/F*_*m*_′ by >20%.

	IC_10_ parent	IC_10_ aged	IC_20_ parent	IC_20_ aged
*Symbiodinium sp*.				
Diuron	0.64 (0.57–0.71)	0.17 (0.12–0.24)	0.68 (0.58–0.79)	0.34 (0.32–0.36)
Atrazine	8.6 (8.0–9.3)	7.2 (6.5–8.0)	14 (13–15)	12 (11–13)
Ametryn	0.47 (0.43–0.51)	0.43 (0.41–0.46)	0.82 (0.77–0.87)	0.80 (0.76–0.83)
Hexazinone	5.3 (4.5–6.7)	10 (8.7–12)	12 (11–14)	17 (16–19)
Simazine	21 (19–23)	22 (20–24)	34 (32–36)	35 (32–38)
DEA	103 (93–115)	—	218 (203–233)	—
*Dunaliella sp*.				
Diuron	1.02 (0.94–1.10)	0.63 (0.58–0.69)	1.74 (1.65–1.84)	1.27 (1.19–1.34)
Atrazine	12 (11–14)	5.9 (5.0–7.1)	18 (16–20)	11 (10–12)
Ametryn	0.70 (0.65–0.74)	0.75 (0.72–0.78)	1.23 (1.17–1.28)	1.35 (1.31–1.38)
Hexazinone	16 (15–17)	18 (17–20)	10 (9.2–11)	10 (9.0–11)
Simazine	31 (29–33)	31 (26–38)	17 (15–18)	16 (14–18)
DEA	157 (142–173)	—	310 (290–332)	—

**Figure 2:**
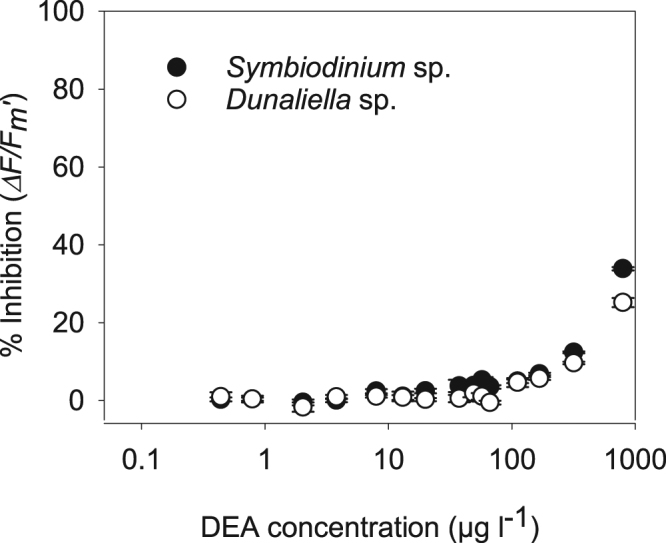
Concentration-response curves for the atrazine transformation product DEA to microalgae. Inhibition of *∆F/F*_*m*_′ (% relative to control) for *Symbiodinium* sp. and *Dunaliella* sp.

In order to assess the toxicity of a subset of the multiple transformation products detected (see below) we selected for toxicity testing three pure transformation products that were commercially available in adequate quantities. *Symbiodinium* sp. and *Dunaliella* sp. were exposed to pure solutions of diuron’s transformation product 3,4-DCA at concentrations up to 273 µg l^−1^, but no significant inhibition of ∆*F/F*_*m*_′ was observed at that concentration^36^. Minor inhibition (<8%) was observed by atrazine’s transformation product DIA at high concentrations of 458 µg l^−1^ for both algal species and this was significant for *Dunaliella* sp. (ANOVA, p < 0.05). Inhibition (>9–35%) by DEA was evident at 84 µg l^−1^ for *Symbiodinium* sp. and at 56 µg l^−1^ for *Dunaliella* sp. (Fig. 2 and SOM Table 6). The inhibition by DEA was greater than 20% at high concentrations, and IC_20_ and IC_10_ values were derived for both microalgae species (Table 2).

### Toxicity of aged herbicide solutions to prawns

**Figure 3:**
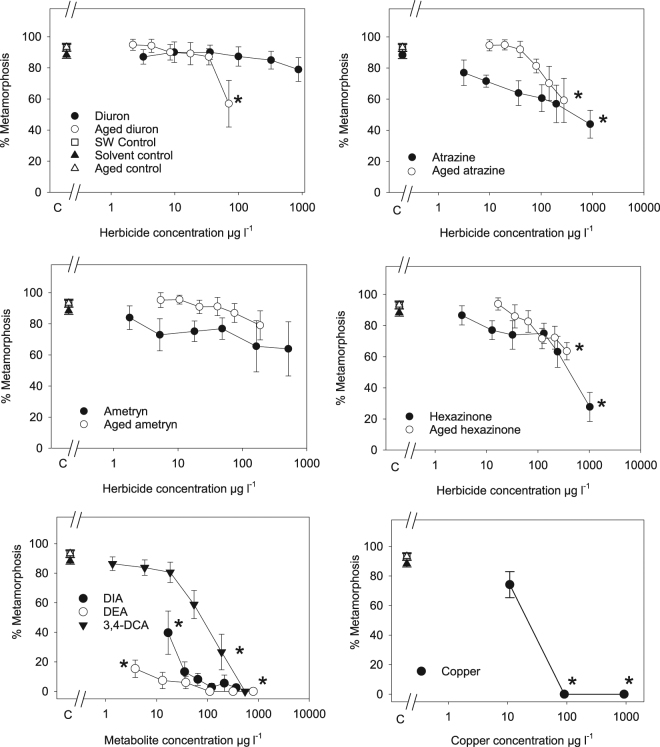
Concentration response relationships for herbicides and their transformation products to prawn larvae. Metamorphosis (%) for larval prawns in the presence of parent herbicides, aged herbicide solutions, and copper. *Indicates significant decrease in metamorphosis in comparison to solvent control samples (p < 0.05, ANOVA SOM Table 7). C = controls. The reduced metamorphosis in the DIA and DEA treatments were all significantly different than controls.

Prawn larval metamorphosis in control treatments was high (88.3–93.3%) and this was not different between seawater alone and the solvent control and aged water controls (Fig. 3, SOM Table 7). Three concentrations of copper were tested as a reference in order to bracket the estimated 24 hr effect concentrations (IC_50s_) from other studies on larval prawns^44–46^. Copper inhibited metamorphosis as expected between 11 and 91 µg l^−1^ (Fig. 3, Table 3). The pure herbicides affected the prawn metamorphosis significantly only at the highest concentrations applied, with the exception of diuron and ametryn which did not affect metamorphosis (Fig. 3, Table 3). The effects herbicides, aged herbicide solutions and the transformation products (3,4-DCA, DIA and DEA) on prawn larvae did not yield inhibition data that was suitable for generating IC_X_ values using non-linear functions (Fig. 3, Table 3). Instead we derived no observed effect concentrations (NOEC) and lowest observed effect concentrations (LOEC) as the next preferred option for assessing toxicity.

**Table 3:** Effects of herbicides and their transformation products on the success of prawn larval metamorphosis. NOEC = no observed significant effect concentration and LOEC = lowest observed significant effect concentration. Significantly different from solvent control when ANOVA p < 0.05.

	NOEC (µg l^−1^)	LOEC (µg l^−1^)	F (df)	P value
Diuron	874	>874	0.53 (6,47)	0.7819
Aged Diuron	34	71	3.3 (6,47)	0.0096
3,4-DCA	54	188	16.6 (6,45)	0.000
Atrazine	197	899	3.97 (6,47)	0.0032
Aged Atrazine	143	278	3.34 (6,47)	0.0090
DIA	0	3.5	23.9 (6,47)	0.000
DEA	0	3.8	63.6 (6,47)	0.000
Ametryn	517	>517	0.57 (6,47)	0.7493
Aged Ametryn	188	>188	0.9 (6,47)	0.5044
Hexazinone	242	1026	4.60 (6,44)	0.0013
Aged Hexazinone	213	366	3.70 (6,46)	0.0051
Copper reference	11	91	98.0 (3,29)	0.000

The aged herbicide solutions also inhibited metamorphosis significantly only at the highest concentrations applied and the aged ametryn solution did not inhibit metamorphosis at the highest concentration tested (Table 3). While, there appeared to be differences in inhibition between parent herbicides and aged solutions in Fig. 3, most of the low-medium concentrations applied did not cause significant inhibition (See SOM Table 8 for concentrations of aged solutions containing the parent compound and transformation products). The only exception was aged diuron solutions which inhibited larval metamorphosis at lower diuron concentrations (71 µg l^−1^) than the pure diuron solutions (>874 µg l^−1^), indicating a contribution of transformation products to the toxicity.

The metamorphosis of larval prawns was sensitive to transformation products of atrazine, with both DIA and DEA significantly inhibiting metamorphosis at all concentrations tested (3.5 to 917 µg l^−1^) (Fig. 3, Table 3). The highest treatment of aged atrazine contained 278 µg l^−1^ atrazine and also 5.1 and 36.3 µg l^−1^ DIA and DEA respectively, sufficient to affect metamorphosis (Fig. 3). Larval metamorphosis was far less sensitive to diuron’s transformation product 3,4-DCA with metamorphosis affected only in the highest treatments of 189 and 547 µg l^−1^ (Fig. 3, Table 3). However, the contribution of the transformation products to the overall toxicity of the aged diuron solution is unclear as the highest treatment of aged herbicide (71 µg l^−1^ diuron) contained two other transformation products at higher concentrations than both diuron and 3,4-DCA (Table 4). The contribution of other transformation products to toxicity is uncertain as they were not available in quantities required for toxicity testing.

**Table 4:** Herbicide concentrations (µg l^−1^) at days 0 and day 330. Includes transformation products and concentrations which were measured and estimated. (See SOM Tables 3 and 4 for identification and quantification details).

Parent herbicide	Time (d)	Parent herbicide concentration	% degradation	Transformation product concentration		
Diuron				3,4- Dichloroaniline	DCPU	DCPMU
	0	967		BDL	BDL	BDL
	330	71	93%	2.1	85	236
				Desethyl atrazine	Desisopropyl Atrazine	Atrazine hydroxy
Atrazine	0	773		BDL	BDL	BDL
	330	278	64%	36	5.1	30
				Ametryn hydroxy	Ametryn desethyl	Ametryn desisopropyl
Ametryn	0	429		BDL	BDL	BDL
	330	189	56%	188	23	BDL
				Hexazinone oxy	Hexazinone hydroxy	Hexazinone desmethyl
Hexazinone	0	871		BDL	BDL	BDL
	330	366	58%	26	5.4	91
				Desethyl simazine	Simazine hydroxy	Simazine amine
Simazine	0	1024		BDL	BDL	BDL
	330	384	63%	79	45	31

### Herbicide degradation and identification of transformation products

After 330 d the experiment was stopped as our previous study indicated that considerable proportions of the parent herbicides should have degraded by this time^8^. Between 56–93% of each of the parent herbicides had degraded by the end of the experiment (Table 4), generating a series of “aged” herbicide solutions that we expected to be rich in transformation products. Transformation products (Table 4), where possible, were confirmed by comparison of retention time and spectra with available standards (SOM Table 2). Other potential transformation products, for which no standards were available, were identified by comparison of fragmentation patterns with literature data. The fragments used to quantify transformation products are provided in SOM Tables 3 and 4. ABSciex Multiquant software was used for quantification of transformation products by comparison to a five-point calibration curve using analytical standards when available or from response factors of the parent compound (modified as indicated) when standards were not available (SOM Table 2).

## Discussion

Assessing the potential risks posed by herbicides and insecticides to aquatic environments requires an understanding of concentrations in the environment as well as the impacts of the mixtures of these toxicants to relevant species. However, the majority of monitoring programs and risk assessments consider only the parent compounds and at most minor subset of transformation products^2,3,47,48^. There is increasing interest in including transformation products as part of the risk assessment processes, as these compounds are often toxic and can occur in substantial concentrations in the environment^28,49^. The present study aimed to determine whether or not naturally produced herbicide transformation products are likely to contribute to toxicity where degradation has taken place. In most of the aged herbicide mixtures, the transformation products did not add to the toxicity of PSII herbicides in microalgae. However, aged diuron mixtures were significantly more toxic to *Symbiodinium* and *Dunaliella* sp. than was explained by the measured diuron alone. This additional toxicity was likely due to the contribution of transformation products, often not monitored in the environment. Furthermore, the toxicity of aged diuron solutions to prawn larvae was greater than that of diuron alone, and the herbicide degradation products 3,4-DCA, DEA and DIA were all more toxic to prawn larvae than their parent herbicides diuron and atrazine.

The acute microalgal toxicity assays allowed the rapid and sensitive assessments of PSII toxicity across a wide range of concentrations^16,50^. The pure herbicides inhibited *∆F/F*_*m*_′ in these assays at similar concentrations to previous microalgal toxicity studies^16,50^. The IC_10_ values for ametryn and diuron were also within the range of PSII herbicide concentrations detected in waters flowing into the nearshore coastal zones of the GBR during flood plumes^47^. Concentration-response curves (IC_X_s) of aged diuron solutions were different to the parent herbicides for *Symbiodinium* sp. and *Dunaliella* sp. where the aged diuron solutions had an IC_50_s 33% and 7% lower (respectively) than that calculated for diuron alone. This increased toxicity of the aged diuron solution was not likely not due to the commonly occurring breakdown product 3,4-DCA, which in pure form did not inhibit *∆F/F*_*m*_′ in these species at concentrations up to 273 µg l^−1^ (SOM Tables 6). 3,4-DCA was also previously demonstrated to exert little toxicity on a range of other marine microalgae species^16^. Other diuron transformation products (i.e. DCPU and DCPMU), that we were able to detect but not test for toxicity, may have been responsible for the additional toxicity of the aged diuron solution (SOM Table 5). Low but significant effects on *∆F/F*_*m*_′ in microalgae were observed from the transformation products of atrazine DEA (both *Symbiodinium* sp. and *Dunaliella* sp.) and DIA (*Dunaliella* sp.) (Table 2 and SOM Table 6). DEA was over 10-fold less toxic to both algal species than atrazine (Table 2) and the IC_10_ values for *Symbiodinium* sp. and *Dunaliella* sp. of 104 and 157 µg l^−1^ DEA respectively were similar to IC_10_ values reported previously for *Navicula* sp. (111 µg l^−1^), *Nephroselmis pyriformis* (26.6 µg l^−1^), *Phaeodactylum tricornutum* (46 µg l^−1^) and *Cylindrotheca closterium* (102 µg l^−1^)^16^. Moderate concentrations of DIA and DEA (<40 µg l^−1^) were detected in the aged atrazine solutions, but below their toxicity threshold (IC_10_) concentrations. Their lack of contribution to the toxicity of aged atrazine solutions to the microalgae was confirmed with nearly identical IC_50_s calculated from concentration-response curves for atrazine and its aged mixture. The relatively high concentrations of DIA and DEA that inhibited *∆F/F*_*m*_′ in these species were also considerably higher than concentrations detected in the nearshore GBR region^28,47^.

Invertebrates are generally insensitive to herbicides unless exposed at the mg l^−1^ concentration range^51^. The moderate inhibition of prawn larval metamorphosis to some of the parent herbicides could potentially be due to a non-specific (baseline effect) toxicity^36,52^. The response of prawn metamorphosis to the herbicides and their aged solutions was variable in comparison with the effects on photosynthesis in microalgae. This is not surprising as the parent herbicides were specifically designed to bind to the D1 protein in Photosystem II and inhibit electron transport which was measured using PAM fluorometry. It is also not surprising that some transformation products of these herbicides may be (by chance) more toxic to non-phototrophic species, as was observed for the aged diuron solution (containing transformation products) which was more toxic to larval metamorphosis than diuron itself. The diuron transformation product 3,4-DCA was only moderately toxic to prawn metamorphosis in agreement with previous studies on its toxicity to some invertebrates such as *Gammarus pulex* (NOEC = 60 µg l^−1^), but inconsistent with its low toxicity to *Brachionus plicatilis* and *Brachionus calyciflorus* (LC_50_ values of ~ 60 mg l^−1^)^53^, and its high toxicity to *Daphnia longispina*, and *Simocephalus vetulus* (both 1 µg l^−1^ after 17 d)^54^. The low concentration of 3,4-DCA measured in the degradation mixture (maximum ~ 2 µg l^−1^) did not account for toxicity to prawn larvae by the aged diuron solution. This additional toxicity was therefore again likely to have been due to other transformation products^36^.

An unexpected finding was the sensitivity of prawn larvae to the individually tested transformation products of atrazine. DIA and DEA were strong inhibitors of naupliar development; however, we regard this this information as preliminary for two reasons. Firstly, an additional experiment should be performed as the data was generated from a single series of tests (as the prawn species used in the experiment spawn only for a limited period there was no opportunity for repeated trials). Secondly, the DEA and DIA detected in the highest aged atrazine solution ought to have caused greater inhibition than was observed (~59% inhibition at a total DIA and DEA concentration greater than 40 µg l^−1^). In a previous study, the DIA and DIA were reported to be far less toxic to invertebrates with 96-hour LC_50_ values for *Hyalella azteca* and *Diporeia* spp. of >3000 µg l^−1^ for compounds^55^. While most of the aged solutions inhibited larval development at concentrations greater than expected in the field, this assay has the potential to identify toxicity of transformation products (including DIA and DEA) to non-target invertebrates and therefore merits additional development and assessment.

The approach used in this study to obtain naturally aged herbicide material enabled us to explore (in a practical way) the likelihood of multiple (known and unknown) transformation products that may contribute to herbicide toxicity^36^. The methodology also enabled us to identify a suite of likely transformation products that could be monitored in the field. Structures of these transformation products were identified from mass spectral databases, reports of transformation products in the literature (e.g. hexazinone hydroxy and oxy), or were assigned tentative structures as postulated from fragmentation (QTOF spectra) data (e.g. simazine amine)^36^. Further confirmation of simazine amine is required through isolation and interrogation via additional structural platforms (e.g. NMR). It is also likely that the profiles and total concentrations of these transformation products will change over time as previously reported for the degradation of diuron and atrazine^8^. The degradation of four of the five herbicides by 56–64% at 330 d in the aged solutions should have resulted in appreciable proportions of transformation products^8^ for toxicity comparisons with pure parent compounds. Diuron degraded more rapidly (by 93% over this period) than reported previously^8^ and an earlier sampling point may have yielded higher concentrations of transformation products of this herbicide. This approach provides a framework or structure for future work that includes assessing in more detail the temporal changes in transformation product profiles and incorporating transformation product toxicities in more comprehensive risk assessments, especially for emerging herbicide compounds to relevant species (tropical marine algae, invertebrates).

Few toxicological studies have included the impact of herbicide mixtures containing potentially toxic transformation products^16,56–58^. The transformation products of diuron and atrazine contributed to additional toxicity in microalgae or prawn larvae. And, although transformation products of the other herbicides tested did not appear to contribute to algal toxicity, under other natural conditions, different (or different proportions of) potentially toxic products may be generated, adding to a presently unrecognised environmental risk. Improved confidence in environmental risk assessments therefore requires further experimental degradation data for herbicides (and mixtures of herbicides which are commonly detected)^2,3^ under relevant environmental conditions, in combination with toxicity data to a range of representative species^49^. The current study revealed potentially high toxicity of atrazine’s transformation products DIA and DEA to a non-phototropic organism, highlighting the need to consider the hazard to species that would not be normally considered sensitive to the parent contaminant. The potential for herbicide transformation products to contribute to toxicity in the environment identified here supports the recommendation that the toxicity of emerging compounds and their transformation products to be assessed for their impact in the marine environment^36^.

## Methods

### Herbicide degradation setup

Photosystem II herbicides/transformation product solutions were produced by adding herbicides to seawater within separate 120 l outdoor fibreglass tanks, in the presence of natural coastal sediments (further details can be found in Mercurio 2016)^36^. Water samples were taken for application in toxicity tests after 330 d as our previous study indicated that considerable proportions of the parent herbicides should have degraded by this time^8^. The tanks used were previously described in Mercurio *et al*.^8^, with treatment water being partially shaded (70%) and exposed to a maximum of 700 µmol photons m^−2^s^−1^ over the course of the experiment. The application of a natural diurnal light regime and coastal sediment were previously shown to increase the rates of PSII herbicide degradation and represent more natural conditions than those used in standard degradation tests^8,36^. Evaporation was minimised with loose-fitting clear acrylic lids and the water continuously circulated using Turbelle Nanostream pumps^59^. Prior to every sampling period evaporation losses were replenished with equal volumes of MilliQ water. The average temperature was 25.7 °C with a range of 15.6–36.6 °C over the course of the study. Unfiltered coastal seawater and sediments were collected from the intertidal zone adjacent to the Australian Institute of Marine Science (19°16′S, 147°03′E), Cape Cleveland, QLD. The sediments were prepared one week prior to use by sieving (>2 mm removed) and thorough mixing. Each tank contained 5 kg of sediment. Physical and chemical information on the seawater and sediments used the treatments can be found in SOM Table 1.

### Herbicide addition, sampling and analysis

The 20 tanks included replicates for control seawater (n = 4) and the PSII herbicides diuron (n = 4), atrazine (n = 3), simazine (n = 3), hexazinone (n = 3), and ametryn (n = 3). Herbicide treatments and replicates were randomized among tanks. The herbicides were purchased from Sigma Aldrich (>95% purity) and were introduced at ~1 mg l^−1^ to enable direct chemical and toxicological testing of the herbicide transformation product mixtures without additional concentration steps. Ethanol was used as a carrier solvent to assist in solubility (final concentration less than 0.01% v/v). The same concentration was used in the controls. Sample collection, internal standard addition, and analytical techniques (HPLC-MS/MS using an AB/Sciex API5500Q mass spectrometer equipped with an electrospray interface and coupled to a Shimadzu Prominence HPLC system) were as previously reported^7,36^. Samples were periodically monitored for the parent herbicide and common herbicide transformation products (i.e. diuron degrading to 3,4-DCA; atrazine degrading to DEA and DIA, see SOM Table 2 for details of transformation products detected) over the course of the regular sampling. Individual replicates were analysed using HPLC/TripleTOF mass spectrometry for possible transformation products as described in Mercurio 2016^36^. Briefly, 0.2 µm-filtered samples were directly injected into an ABSciex API5600+ Triple TOF mass spectrometer (ABSciex, Concord, Ontario, Canada) equipped with an electrospray (TurboV) interface coupled to a Shimadzu Nexera HPLC system (Shimadzu Corp., Kyoto, Japan). Separation was achieved using a 4 micron 50 × 2.0 mm Phenomenex Synergi Fusion RP column (Phenomenex, Torrance, CA) run at 45 °C, and a flow rate of 0.4 mL min^−1^ with a linear gradient starting at 8% B for 0.5 minutes, ramped to 100% B in 8 minutes then held at 100% for 2.0 minutes followed by equilibration at 8% B for 2.5 minutes (A = 1% methanol in HPLC grade water, B = 95% methanol in HPLC grade water, both containing 0.1% acetic acid). The mass spectrometer was operated in positive ion SWATH mode. Briefly this mode comprises a TOF scan of 50 millisecond duration followed by small segments of the mass range being transmitted through the quadrupole, fragmented in the collision cell and full TOF mass spectra taken of the transformation products. Data from these experiments was examined using the Masterview software (ABSciex).

Potential transformation products identified using TOF mass spectrometry and other potential transformation products identified from literature were then re-examined by HPLC/ triple quadrupole mass spectrometry using multiple reaction monitoring^36^. Product ions used were as identified from QTOF data or from literature references and parameters such as collision energy optimised by repeated injections of samples for compounds detected. Standards were obtained for some of these compounds and all samples re run (method details as for parent compound analysis with extra transformation products as per SOM Table 2). Samples were analysed via direct injection using HPLC-MS/MS with multiple reaction monitoring (SOM Tables 3/4), with a standard calibration at beginning and end, and additional quality control standards run every 10 samples^7^.

Herbicide transformation product mixtures containing the most aged parent herbicide were chosen for the toxicity experiments. For toxicity experiments, all dilutions of all herbicide solutions were made using the control seawater from the 330 d experiment. Control treatments for each toxicity assay included: 0.2 µm filtered fresh seawater (FSW), 0.2 µm filtered solvent control seawater (SC), and 0.2 µm filtered seawater sampled from control tanks after 330 d (=aged control).

### Microalgal assays

Microalgae play a critical role in the marine food web and have been used in a number of sensitive assays for the assessment of toxicity of both herbicides and their transformation products^13,16,60^. High throughput 96 well plate designs allow for increased replication and a wide range of concentrations especially when paired with pulse amplitude modulation (PAM) fluorometry^50,61^. PAM fluorometry measures chlorophyll fluorescence and can be used to calculate inhibitions of effective quantum yield *∆F/F*_*m*_′, which is proportional to reduced photosynthetic efficiency^62^ and growth in microalgae^63^ and can be plotted against toxicant concentrations to derive concentrations that inhibit *∆F/F*_*m*_′ by 10, 20 and 50% (IC_10_, IC_20_ and IC_50_). In the present study the green algae (*Dunaliella* sp.) and coral symbiont (*Symbiodinium* sp.) were exposed to herbicides and aged herbicide solutions for 24 h in a 96-well plate format^14^. A Maxi-Imaging-PAM (I-PAM) (Walz, GmbH Germany) was used to measure inhibition of *∆F/F*_*m*_′ using settings provided previously^36^ and below.

*Symbiodinium* cells were isolated from coral by air blasting branches of *Acropora tenuis* colonies collected (collected under the permit G10-33440.1) at 2–5 m depth in Nelly Bay, Magnetic Island, GBR^36^. *Symbiodinium* cells were inoculated into sterile IMK growing media, the culture purified, DNA extracted, and Clade C1 identified as published previously^64,65^. Cultures were maintained at 26 °C, 60 µE PAR, 14:10 light:dark photoperiod inside environmental chambers (Steridium e500). The *Dunaliella* sp. (CS-353) (Chlorophyceae) was obtained from the CSIRO Collection of Living Microalgae (CCLM). The algae was subcultured and grown in F2 media. For the toxicology assays, an exponentially growing culture was employed^63^ and density adjusted by hemocytometry under 10× magnification^36^. Day 7 sub-cultures were used throughout the experiment.

Herbicide solutions were delivered into each treatment plate using a Perkin Elmer Janus liquid handling system. Each well of the black 96-well plates (Perkin Elmer) received 100 µl herbicide solution as well as 100 µl of algal suspension (added via multi-channel pipette) and the solution was gently mixed. The 96 well plate positions included controls (as described above; filtered seawater controls (n = 16), solvent controls (n = 8), aged controls (n = 8)) and randomised treatment samples across a concentration range targeting IC_50_ values (n = 4 at each concentration)^36^. Each plate included a 3 µg l^−1^ positive diuron control (n = 4) to confirm consistent sensitivities among the replicate algal subcultures. Treatments positions were randomised across the plates in duplicate^50,61,66^. *Symbiodinium* culture plates were incubated over a 12:12 h light:dark cycle at 26 °C and 60–70 µmol photons m^−2^ s^−1^ and *Dunaliella* sp. Plates incubated at: 26 °C and 130 µmol photons m^−2^ s^−1^. Microalgae were exposed for 24 h prior to PAM measurements. Samples were subjected to the 50 µmol photons m^−2^ s^−1^ actinic light for 1 min prior to measurement in the Maxi-IPAM (Actinic light = 1, ML = 10, ML frequency = 8, gain = 2 and damping = 1).

### Prawn larvae assay

The giant tiger prawn (*Penaeus monodon*) can be found throughout the tropical Indo-Pacific region and can be obtained from aquaculture facilities and has a well-described life cycle. The aim of the larval prawn assay was to determine whether the herbicides and transformation products inhibit early naupliar development. Prawn eggs hatch into their first larval stage (nauplii), typically 12–15 h after spawning^67^. During development the nauplii are lecithotrophic, and over the course of the next 36 h, nauplii pass through 6 sub-stages before metamorphosing into protozoae^67,68^.

Freshly hatched nauplii were harvested by light attraction over 30 min, and washed and aerated for 10 min with seawater, and transported to AIMS in Townsville, Queensland. Pilot experiments demonstrated normal development without feeding at stocking density up to 1000 nauplii l^−1^ and we subsequently cultured at conservative stocking densities of 75–150 nauplii l^−1 ^^36^. The toxicity assays were performed in incubator shakers (set to 30 °C) under very low light and with gentle shaking to prevent individuals adhering to the side of experimental containers.

The larval stock was gently concentrated via reverse-gravity filtration to a higher density before dispensing 8–10 individuals per 20 ml glass scintillation vials. Additional positive control treatments in the form of 4 concentrations of copper II chloride solution (2 to 900 µg l^−1^ Cu), were included for test validation^36^. The static assay was terminated after 24 h and 10% seawater formalin was added as a preservative for later microscopy. Metamorphosis was considered successful when nauplii had developed into protozoea.

### Data handling and analysis

The inhibition of photosynthetic efficiency (*∆F/F*_*m*_′) and metamorphosis was calculated as a percentage relative to control where Inhibition (%) = 100 × [(Control − Treatment)/Control]. The concentrations that inhibited 10%, 20% and 50% of photosynthetic yield (IC_10_, C_20_, and IC_50_) was calculated from concentration-response curves (four-parameter logistic models) fitted to the % inhibition and log transformed concentration data of each treatment using the program GraphPad Prism (v6, San Diego, USA). The model was constrained by applying a lower limit of 0% inhibition and all curves were tested for normality of the residuals and a replicate test was applied to assess the goodness of fit^36^. The probability that IC_X_ values generated by the logistic curves were statistically different between parent and aged herbicide solutions was tested by applying the F test in Graph Pad Prism v6. IC_X_s were considered different when p < 0.05. Where inhibition data could not be fitted to logistic curves, one-way analysis of variance (ANOVA) was performed to identify treatments which caused significant (p < 0.05) inhibition comparison with control treatments (NCSS v9, Utah, USA). The larval prawn experimental data was arcsine square root transformed prior to statistical analysis.

### Data Availability Statement

The datasets generated during and/or analysed during the current study are available from the corresponding author on reasonable request.

## Electronic supplementary material

Supplementary Information
