# Supplementary material for: Contribution of transformation products towards the total herbicide toxicity to tropical marine organisms
Source: Sci Rep. 2018 Mar 19;8:4808. doi: 10.1038/s41598-018-23153-4 (PMC5859250; doi:10.1038/s41598-018-23153-4)
Supplement: Supplementary file 1 — Supplementary Information [file 41598_2018_23153_MOESM1_ESM.doc]

# Contribution of transformation products to the total herbicide toxicity in tropical marine organisms

Philip Mercurio^1,2*^, Geoff Eaglesham^2^, Stephen Parks^3^, Matt Kenway^1^, Victor Beltran^1^, Florita Flores^1^, Jochen F. Mueller^2^, Andrew P. Negri^2^

1. Australian Institute of Marine Science, Townsville, Queensland, Australia

2. The University of Queensland, Queensland Alliance for Environmental Health Sciences, Coopers Plains, Queensland, Australia

3. James Cook University, Townsville, Queensland, Australia

*Corresponding author.

email: pmercurio@gmail.com

**SOM Table 1: Physical and chemical information for the seawater used in open tanks, averages n=3.**

| **Parameter** | **Value** | **Units** |
| --- | --- | --- |
| pH | 8.2 |  |
| POC | 0.35 | mg l^-1^ |
| N | 0.04 | mg l^-1^ |
| NPOC/DOC (TOC) | 1.44 | mg l^-1^ |
| DIC | 26.0 | mg l^-1^ |
| NH_4_ | 0.61 | µmol l^-1^ |
| PO_4_ | 0.2 | µmol l^-1^ |
| NO_2_ + NO_3_ | 1.32 | µmol l^-1^ |
| NO_2_ | 0.14 | µmol l^-1^ |
| Si | 8.57 | µmol l^-1^ |
| TDP | 0.28 | µmol l^-1^ |
| TDN | 9.55 | µmol l^-1^ |
| Salinity | 34 | ‰ |
| Sediment TC | 1.04 | % |
| Sediment TOC | 0.46 | % |
| Sediment TN | 0.02 | % |
| Sediment mean particle size | 218.5 | µm |

SOM Table 2: Transformation products measured in aged herbicide samples. Bold indicates quantified against an analytical standard, other metabolites were quantified using response factors as indicated. Acquisition parameters including precursor mass can be found in SOM Table 3.

| Herbicide | Transformation products |  | Response factors calculated from |
| --- | --- | --- | --- |
| Diuron | **3-(3,4-dichlorophenyl)-1-methylurea** | **DCPMU** | Analytical standard |
|  | **3,4-dichlorophenylurea** | **DCPU** | Analytical standard |
|  | **3,4- dichloro aniline** | **3,4-DCA** | Analytical standard |
| Atrazine | **hydroxy atrazine** |  | Analytical standard |
|  | **desethyl atrazine** | **DEA** | Analytical standard |
|  | **desisopropyl atrazine** | **DIA** | Analytical standard |
| Simazine | **hydroxy simazine** |  | Analytical standard |
|  | **desethyl simazine** | **= desethyl atrazine DEA** | Analytical standard |
|  | simazine amine* |  | Simazine |
| Ametryn | **hydroxy ametryn** |  | Analytical standard |
|  | ametryn desethyl |  | Ametryn** |
|  | ametryn desisopropyl |  | Ametryn** |
| Hexazinone | hexazinone desmethyl |  | Hexazinone** |
|  | hexazinone oxy |  | hexazinone |
|  | hexazinone hydroxy |  | hexazinone |

* structure suggested from TripleTOF data

** response modified by relative response of the equivalent atrazine metabolite to atrazine

SOM Table 3: ABSciex 5500 QTrap aquisition parameters. Q1: precursor mass, Q3: fragment mass (1 = quantifier, 2 = qualifier), Dwell: dwell time in milliseconds (MRM) or retention time of analyte (sMRM), DP: declustering potential, EP: entrance potential, CE: collision energy, CXP: collision cell exit potential. Note: Atrazine hydroxy and Ametryn hydroxy have the same structure as do their desethyl and desisopropyl forms. Desisopropyl atrazine and desethyl simazine also have the same structure.

| Q1 | Q3 | Dwell/RT | ID | DP | EP | CE | CXP |
| --- | --- | --- | --- | --- | --- | --- | --- |
| 202.1 | 132 | 15 | Simazine 1 | 70 | 10 | 27 | 14 |
| 202.1 | 124 | 15 | Simazine 2 | 70 | 10 | 25 | 14 |
| 184.1 | 114 | 15 | Simazine hydroxy 1 | 70 | 10 | 27 | 12 |
| 184.1 | 69 | 15 | Simazine hydroxy 2 | 70 | 10 | 37 | 10 |
| 183.1 | 113 | 15 | Simazine amine 1 | 70 | 10 | 27 | 12 |
| 183.1 | 68 | 15 | Simazine amine 2 | 70 | 10 | 42 | 10 |
| 174.1 | 104 | 15 | Simazine desethyl 1 | 70 | 10 | 32 | 10 |
| 174.1 | 96 | 15 | Simazine desethyl 2 | 70 | 10 | 25 | 9 |
| 233.05 | 72 | 15 | Diuron 1 | 70 | 10 | 40 | 12 |
| 233.05 | 46 | 15 | Diuron 2 | 70 | 10 | 38 | 12 |
| 162 | 127 | 15 | 3,4-dichloroaniline 1 | 70 | 10 | 28 | 12 |
| 162 | 74 | 15 | 3,4-dichloroaniline 2 | 70 | 10 | 68 | 12 |
| 199.02 | 72 | 15 | mCPDMU 1 | 60 | 10 | 32 | 10 |
| 199.01 | 46 | 15 | mCPDMU 2 | 60 | 10 | 29 | 9 |
| 205.03 | 127 | 15 | DCPU 1 | 60 | 10 | 38 | 13 |
| 205.01 | 162 | 15 | DCPU 2 | 60 | 10 | 21 | 15 |
| 219.01 | 127 | 15 | DCPMU 1 | 60 | 10 | 35 | 13 |
| 219.02 | 162 | 15 | DCPMU 2 | 60 | 10 | 20 | 15 |
| 216.1 | 174 | 15 | Atrazine 1 | 70 | 10 | 25 | 16 |
| 216.1 | 96 | 15 | Atrazine 2 | 70 | 10 | 34 | 12 |
| 188.1 | 146 | 15 | Desethyl Atrazine 1 | 70 | 10 | 24 | 17 |
| 188.1 | 104 | 15 | Desethyl Atrazine 2 | 70 | 10 | 35 | 12 |
| 253.2 | 171 | 15 | Hexazinone 1 | 70 | 10 | 22 | 16 |
| 253.2 | 71 | 15 | Hexazinone 2 | 70 | 10 | 40 | 8 |
| 267.1 | 171 | 15 | Hexazinone oxy 1 | 70 | 10 | 27 | 16 |
| 267.1 | 71 | 15 | Hexazinone oxy 2 | 70 | 10 | 42 | 8 |
| 269.1 | 171 | 15 | Hexazinone hydroxy 1 | 70 | 10 | 27 | 16 |
| 269.1 | 71 | 15 | Hexazinone hydroxy 2 | 70 | 10 | 42 | 8 |
| 239.1 | 157 | 15 | Hexazinone desmethyl 1 | 70 | 10 | 27 | 16 |
| 239.1 | 71 | 15 | Hexazinone desmethyl 2 | 70 | 10 | 42 | 8 |
| 228.2 | 186 | 15 | Ametryn 1 | 70 | 10 | 26 | 16 |
| 228.2 | 116 | 15 | Ametryn 2 | 70 | 10 | 36 | 14 |
| 200.1 | 158 | 15 | Ametryn desethyl 1 | 70 | 10 | 27 | 12 |
| 200.1 | 68 | 15 | Ametryn desethyl 2 | 70 | 10 | 50 | 12 |
| 186.1 | 144 | 15 | Ametryn desisopropyl 1 | 70 | 10 | 27 | 14 |
| 186.1 | 116 | 15 | Ametryn desisopropyl 2 | 70 | 10 | 32 | 10 |
| 198.11 | 156 | 15 | Ametryn hydroxy 1 | 70 | 10 | 25 | 14 |
| 198.11 | 86 | 15 | Ametryn hydroxy 2 | 70 | 10 | 32 | 10 |
| 156.11 | 86 | 15 | Ametryn hydroxy desisopropyl 1 | 70 | 10 | 22 | 10 |
| 156.11 | 114 | 15 | Ametryn hydroxy desisopropyl 2 | 70 | 10 | 22 | 12 |
| 170.11 | 128 | 15 | Ametryn hydroxy desethyl 1 | 70 | 10 | 23 | 12 |
| 170.11 | 86 | 15 | Ametryn hydroxy desethyl 2 | 70 | 10 | 21 | 10 |

SOM Table 4: ABSciex 5600 TripleTOF acquisition parameters.

| SWATH | 5600+ TripleTOF | ABSciex |  |  |
| --- | --- | --- | --- | --- |
|  | Total cycle time 0.82 seconds |  |  |  |
| Q2 | Dwell (milliseconds) | Collision Energy  (milli volts) | Collision Energy Spread | TOF  (range 10-1000) |
| 100 - 1000 | 50 | no collision energy |  | full scan |
| 100 - 150.5 | 30 | 35 | 15 | full scan |
| 149.5 - 170.5 | 30 | 35 | 15 | full scan |
| 169.5 - 185.5 | 30 | 35 | 15 | full scan |
| 184.5 - 197.5 | 30 | 35 | 15 | full scan |
| 196.5 - 209.5 | 30 | 35 | 15 | full scan |
| 208.5 - 221.5 | 30 | 35 | 15 | full scan |
| 220.5 - 233.5 | 30 | 35 | 15 | full scan |
| 232.5 - 245.5 | 30 | 35 | 15 | full scan |
| 244.5 - 257.5 | 30 | 35 | 15 | full scan |
| 256.5 - 269.5 | 30 | 35 | 15 | full scan |
| 268.5 - 281.5 | 30 | 35 | 15 | full scan |
| 280.5 - 293.5 | 30 | 35 | 15 | full scan |
| 292.5 - 305.5 | 30 | 35 | 15 | full scan |
| 304.5 - 317.5 | 30 | 35 | 15 | full scan |
| 316.5 - 329.5 | 30 | 35 | 15 | full scan |
| 328.5 - 341.5 | 30 | 35 | 15 | full scan |
| 340.5 - 353.5 | 30 | 35 | 15 | full scan |
| 352.5 - 365.5 | 30 | 35 | 15 | full scan |
| 364.5 - 377.5 | 30 | 35 | 15 | full scan |
| 376.5 - 389.5 | 30 | 35 | 15 | full scan |
| 385.5 - 398.5 | 30 | 35 | 15 | full scan |
| 400.5 - 413.5 | 30 | 35 | 15 | full scan |
| 412.5 - 500.5 | 30 | 35 | 15 | full scan |
| 499.5 - 1000 | 30 | 35 | 15 | full scan |

SOM Table 5: Concentrations (µg l^-1^) of parent and transformation products in aged solutions for all concentrations used in microalgae experiment.

|  | Diuron | 3,4-dichloroaniline | DCPU | DCPMU | mCPDMU |
| --- | --- | --- | --- | --- | --- |
| Aged Diuron | 0.10 | BDL | BDL | BDL | BDL |
| Aged Diuron | 0.47 | BDL | 0.60 | 1.72 | BDL |
| Aged Diuron | 0.52 | BDL | 0.60 | 1.88 | BDL |
| Aged Diuron | 1.09 | BDL | 1.31 | 3.95 | BDL |
| Aged Diuron | 2.15 | BDL | 2.48 | 7.67 | BDL |
| Aged Diuron | 2.16 | BDL | 2.58 | 7.83 | BDL |
| Aged Diuron | 2.33 | BDL | 2.69 | 8.24 | BDL |
| Aged Diuron | 4.23 | BDL | 4.97 | 15.21 | BDL |
| Aged Diuron | 4.58 | BDL | 5.38 | 16.28 | BDL |
| Aged Diuron | 8.80 | 0.14 | 10.22 | 31.61 | BDL |
| Aged Diuron | 9.07 | 0.19 | 10.71 | 32.37 | BDL |
| Aged Diuron | 17.11 | 0.38 | 19.62 | 59.33 | BDL |
| Aged Diuron | 17.68 | 0.37 | 20.93 | 61.90 | BDL |
| Aged Diuron | 35.29 | 1.07 | 42.26 | 117.98 | BDL |
|  | Atrazine | Desethyl  Atrazine | Desisopropyl  Atrazine | Atrazine hydroxy |  |
| Aged Atrazine | 1.26 | 0.14 | BDL | 0.12 |  |
| Aged Atrazine | 2.47 | 0.28 | BDL | 0.23 |  |
| Aged Atrazine | 4.94 | 0.53 | BDL | 0.45 |  |
| Aged Atrazine | 8.01 | 0.85 | 0.12 | 0.74 |  |
| Aged Atrazine | 9.80 | 1.06 | 0.16 | 0.87 |  |
| Aged Atrazine | 16.14 | 1.72 | 0.25 | 1.44 |  |
| Aged Atrazine | 16.37 | 1.82 | 0.27 | 1.52 |  |
| Aged Atrazine | 19.49 | 2.10 | 0.34 | 1.78 |  |
| Aged Atrazine | 25.10 | 2.77 | 0.43 | 2.32 |  |
| Aged Atrazine | 32.60 | 3.58 | 0.50 | 3.01 |  |
| Aged Atrazine | 40.06 | 4.43 | 0.66 | 3.69 |  |
| Aged Atrazine | 47.55 | 5.48 | 0.80 | 4.48 |  |
| Aged Atrazine | 61.59 | 7.17 | 1.09 | 5.90 |  |
| Aged Atrazine | 71.51 | 8.53 | 1.29 | 7.22 |  |
| Aged Atrazine | 139.00 | 18.12 | 2.55 | 14.94 |  |
|  |  |  |  |  |  |
|  | Ametryn | Ametryn hydroxy | Ametryn  desethyl | Ametryn  desisopropyl |  |
| Aged Ametryn | 0.71 | 0.88 | BDL | BDL |  |
| Aged Ametryn | 0.96 | 1.21 | 0.13 | BDL |  |
| Aged Ametryn | 1.34 | 1.74 | 0.19 | BDL |  |
| Aged Ametryn | 2.11 | 2.73 | 0.29 | BDL |  |
| Aged Ametryn | 2.12 | 2.81 | 0.30 | BDL |  |
| Aged Ametryn | 2.69 | 3.53 | 0.37 | BDL |  |
| Aged Ametryn | 3.33 | 4.20 | 0.45 | BDL |  |
| Aged Ametryn | 4.10 | 5.56 | 0.58 | BDL |  |
| Aged Ametryn | 5.27 | 6.67 | 0.71 | BDL |  |
| Aged Ametryn | 6.05 | 8.48 | 0.88 | BDL |  |
| Aged Ametryn | 8.69 | 11.19 | 1.18 | BDL |  |
| Aged Ametryn | 10.73 | 13.24 | 1.41 | BDL |  |
| Aged Ametryn | 20.52 | 27.12 | 2.93 | BDL |  |
| Aged Ametryn | 37.69 | 52.86 | 5.98 | BDL |  |
|  |  |  |  |  |  |
|  | Hexazinone | Hexazinone oxy | Hexazinone  hydroxy | Hexazinone  desmethyl |  |
| Aged Hexazinone | 0.89 | BDL | BDL | 0.16 |  |
| Aged Hexazinone | 2.22 | 0.10 | BDL | 0.38 |  |
| Aged Hexazinone | 4.35 | 0.20 | BDL | 0.76 |  |
| Aged Hexazinone | 8.41 | 0.39 | BDL | 1.46 |  |
| Aged Hexazinone | 14.42 | 0.66 | 0.14 | 2.55 |  |
| Aged Hexazinone | 32.36 | 1.57 | 0.32 | 6.06 |  |
| Aged Hexazinone | 42.59 | 2.08 | 0.42 | 7.91 |  |
| Aged Hexazinone | 51.27 | 2.60 | 0.55 | 10.17 |  |
| Aged Hexazinone | 60.26 | 3.19 | 0.65 | 12.45 |  |
| Aged Hexazinone | 72.06 | 3.96 | 0.79 | 14.99 |  |
| Aged Hexazinone | 93.08 | 5.17 | 1.09 | 19.94 |  |
| Aged Hexazinone | 106.62 | 6.65 | 1.34 | 23.91 |  |
| Aged Hexazinone | 182.83 | 12.96 | 2.70 | 45.22 |  |

BDL = Below detection limit

SOM Table 6: One way ANOVA results for the effect of transformation products on microalgae *∆F/Fm’*. NOEC = no significant observed effect concentration. LOEC = lowest significant observed effect concentration.  Effects on *∆F/F_m_’* were considered significant when p < 0.05.

| Transformation product | df | F ratio | p | NOEC (µg l^-1^) | LOEC (µg l^-1^) |
| --- | --- | --- | --- | --- | --- |
| *Symbiodinium* sp. |  |  |  |  |  |
| DIA | 15,67 | 1.05 | 0.427 | 458.31 | >458.31 |
| DEA | 15,67 | 133.04 | 0.000 | 55.63 | 83.68 |
| 3,4-DCA | 15,67 | 0.79 | 0.684 | 273.41 | >273.41 |
| *Dunaliella* sp. |  |  |  |  |  |
| DIA | 15,67 | 5.51 | 0.000 | 148.97 | 458.31 |
| DEA | 15,67 | 65.37 | 0.000 | 33.30 | 55.63 |
| 3,4-DCA | 15,67 | 1.04 | 0.433 | 273.41 | >273.41 |

SOM Table 7: One way ANOVA results for the effect of parent herbicides, transformation products and aged herbicide mixtures on larval prawn metamorphosis. NOEC = no significant observed effect concentration. LOEC = lowest significant observed effect concentration.  Effects on metamorphosis were considered significant when p < 0.05.  Control test solutions included seawater control, solvent control, and aged controls.

| Herbicide | df | F ratio | p | NOEC (µg l^-1^) | LOEC (µg l^-1^) |
| --- | --- | --- | --- | --- | --- |
| Controls | 2,35 | 1.47 | 0.2455 |  |  |
| Diuron | 6,47 | 0.53 | 0.7819 | 874 | >874 |
| Aged Diuron | 6,47 | 3.3 | 0.0096 | 34 | 71 |
| Atrazine | 6,47 | 3.97 | 0.0032 | 197 | 899 |
| Aged Atrazine | 6,47 | 3.34 | 0.0090 | 143 | 278 |
| Ametryn | 6,47 | 0.57 | 0.7493 | 517 | >517 |
| Aged Ametryn | 6,47 | 0.9 | 0.5044 | 188 | >188 |
| Hexazinone | 6,44 | 4.6 | 0.0013 | 242 | 1026 |
| Aged Hexazinone | 6,46 | 3.7 | 0.0051 | 213 | 366 |
| DIA | 6,47 | 23.9 | 0.000 | 0 | 3.5 |
| DEA | 6,47 | 63.6 | 0.000 | 0 | 3.8 |
| 3,4-DCA | 6,45 | 16.6 | 0.000 | 54 | 188 |
| Copper (reference toxicant) | 3,29 | 98.0 | 0.000 | 11 | 91 |

SOM Table 8: Herbicide and transformation products concentrations (µg l^-1^) used in the larval prawn experiment.

| Herbicide | Measured |  |  |  |  |
| --- | --- | --- | --- | --- | --- |
|  | Diuron | 3,4-dichloroaniline | DCPU | DCPMU | mCPDMU |
| Aged Diuron | 2.20 |  | 2.61 | 7.99 | BDL |
| Aged Diuron | 4.30 |  | 4.95 | 15.33 | BDL |
| Aged Diuron | 8.47 |  | 9.94 | 30.43 | BDL |
| Aged Diuron | 17.60 | 0.27 | 20.44 | 63.22 | BDL |
| Aged Diuron | 34.21 | 0.76 | 39.23 | 118.65 | BDL |
| Aged Diuron | 70.57 | 2.14 | 84.52 | 235.96 | BDL |
|  | Atrazine | Desethyl  Atrazine | Desisopropyl Atrazine | Atrazine hydroxy |  |
| Aged Atrazine | 9.87 | 1.06 |  | 0.90 |  |
| Aged Atrazine | 19.60 | 2.11 | 0.32 | 1.74 |  |
| Aged Atrazine | 38.98 | 4.21 | 0.67 | 3.55 |  |
| Aged Atrazine | 80.12 | 8.85 | 1.32 | 7.38 |  |
| Aged Atrazine | 143.02 | 17.06 | 2.58 | 14.43 |  |
| Aged Atrazine | 277.99 | 36.25 | 5.10 | 29.88 |  |
|  | Ametryn | Ametryn hydroxy | Ametryn desethyl | Ametryn desisopropyl |  |
| Aged Ametryn | 5.37 | 7.06 | 0.74 | BDL |  |
| Aged Ametryn | 10.54 | 13.34 | 1.43 | BDL |  |
| Aged Ametryn | 21.46 | 26.49 | 2.82 | BDL |  |
| Aged Ametryn | 41.03 | 54.24 | 5.87 | BDL |  |
| Aged Ametryn | 75.39 | 105.73 | 11.95 | BDL |  |
| Aged Ametryn | 188.56 | 188.37 | 23.43 | BDL |  |
|  | Hexazinone | Hexazinone oxy | Hexazinone hydroxy | Hexazinone  desmethyl |  |
| Aged Hexazinone | 16.82 | 0.77 |  | 2.92 |  |
| Aged Hexazinone | 35.55 | 1.59 | 0.35 | 6.44 |  |
| Aged Hexazinone | 64.72 | 3.14 | 0.64 | 12.13 |  |
| Aged Hexazinone | 120.53 | 6.39 | 1.29 | 24.90 |  |
| Aged Hexazinone | 213.25 | 13.29 | 2.68 | 47.81 |  |
| Aged Hexazinone | 365.65 | 25.92 | 5.40 | 90.45 |  |
